# Pink1/Parkin deficiency alters circulating lymphocyte populations and increases platelet-T cell aggregates in rats

**DOI:** 10.21203/rs.3.rs-4431604/v1

**Published:** 2024-05-30

**Authors:** Jane E. Manganaro, Katy Emanuel, Benjamin G. Lamberty, Joseph W. George, Kelly L. Stauch

**Affiliations:** University of Nebraska Medical Center; University of Nebraska Medical Center; University of Nebraska Medical Center; University of Nebraska Medical Center; University of Nebraska Medical Center

**Keywords:** B cells, Energetics, Parkin, Pink1, Platelets, T cells

## Abstract

Parkinson’s disease (PD) is the most common progressive neurodegenerative movement disorder and results from the selective loss of dopaminergic neurons in the substantia nigra pars compacta. Pink1 and Parkin are proteins that function together in mitochondrial quality control, and when they carry loss-of-function mutations lead to familial forms of PD. While much research has focused on central nervous system alterations in PD, peripheral contributions to PD pathogenesis are increasingly appreciated. We report Pink1/Parkin regulate glycolytic and mitochondrial oxidative metabolism in peripheral blood mononuclear cells (PBMCs) from rats. Pink1/Parkin deficiency induces changes in the circulating lymphocyte populations, namely increased CD4 + T cells and decreased CD8 + T cells and B cells. Loss of Pink1/Parkin leads to elevated platelet counts in the blood and increased platelet-T cell aggregation. Platelet-lymphocyte aggregates are associated with increased thrombosis risk, and venous thrombosis is a cause of sudden death in PD, suggesting targeting the Pink1/Parkin pathway in the periphery has therapeutic potential.

## Introduction

Parkinson’s disease (PD) is a prevalent, chronic and progressive neurodegenerative disorder that is clinically characterized by motor symptoms, including bradykinesia, rigidity, resting tremor, and postural instability^1^. Diagnosis is largely based on clinical symptoms, but definitive confirmation of the disease requires pathological examination at autopsy, where progressive degeneration of nigral dopamine (DA) neurons, along with Lewy bodies in surviving neurons, is observed^2^. Currently, therapies only treat PD symptoms, mostly by enhancing DA signaling, which is required for normal movement, but do not slow PD progression or protect against neuronal cell death. Most cases of PD are idiopathic, and there is as yet no diagnostic or predictive molecular marker of disease.

Neurotoxins, such as 1-methyl-4-phenyl-1,2,3,6-tetrahydropyridine (MPTP)^3^, paraquat and rotenone^4^, which cause increased reactive oxygen species (ROS) generation and mitochondrial dysfunction, can induce parkinsonism in humans and animal models. The most widely used rodent model of PD is the 6-hydroxydopamine model in which a unilateral lesion causes asymmetrical presentation of symptoms including degeneration of nigral DA neurons and gait deficits^5,6^. The identification of hereditary forms of PD uncovered gene mutations and variants, such as loss-of-function mutations in Pink1 and Parkin, two proteins involved in mitochondrial quality control^7^, that could be harnessed to create genetic mammalian animal models of PD^8^. Parkin is an E3 ubiquitin ligase, targeting proteins for degradation through the ubiquitin-proteasome system, as well as marking proteins on the outer mitochondrial membrane to target mitochondria for autophagic destruction (mitophagy)^9,10^. Phosphatase and tensin homolog (PTEN)-induced kinase 1 (Pink1) accumulates on the surface of mitochondria in response to stress (e.g. ROS^11^ and mitochondrial depolarization^12,13^) and recruits Parkin to promote the selective degradation of mitochondria. The Pink1/Parkin mitophagy pathway is important for mitochondrial quality control, and mitophagy interruption leads to hyperactivation of inflammatory signaling pathways and chronic systemic inflammation^14^. Increasing evidence implicates a role for immunity and inflammation in the onset and progression of PD^14–16^.

Similar to PD in humans, the Pink1 single knockout (sKO) rat model demonstrates early motor and non-motor deficits^17–19^, nigrostriatal DA loss^20^, and nigral DA neuron loss^17,21,22^. Inconsistencies have been noted in the presence of PD-relevant pathological manifestations among cohorts suggesting incomplete phenotypic penetrance^18,23^. Parkin sKO rats appear resistant to motor impairment and nigrostriatal DA neurodegeneration up to 8 months of age^17^ but do show abnormalities in DA metabolites^20^. We generated a combined Pink1/Parkin double knockout rat (dKO), which display a PD-relevant phenotype, including gait abnormalities and tremor as well as α-synuclein aggregation in the striatum that coincides with loss of nigral neurons^24,25^. Indeed, similar to animal models, the phenotypes of PD are diverse in humans, and characterizing early-onset PD is challenging in humans due to differential patterns of symptom manifestation, inconsistent age of disease onset, and environmental variability. While much research has focused on central nervous system alterations in PD, peripheral contributions to PD pathogenesis are increasingly appreciated. As such, the genetic rat models enable studies into the effect of Pink1 and Parkin KO, single and combined, on peripheral pathogenic mechanisms in a mammalian model that exhibits PD-relevant pathology.

In this study, we further characterized the Pink1 sKO, Parkin sKO, and Pink1/Parkin dKO rats. Comparisons of organ weights uncovered differences between the genotypes. Our findings suggest tissue-specific effects of loss of Pink1 and/or Parkin. We report Pink1/Parkin regulate glycolytic and mitochondrial oxidative metabolism in peripheral blood mononuclear cells (PBMCs) from rats. Pink1/Parkin deficiency induces changes in the circulating lymphocyte populations, namely increased CD4 + T cells and decreased CD8 + T cells and B cells. Loss of Pink1/Parkin leads to elevated platelet counts in the blood and increased platelet-T cell aggregation. In PD, it is becoming clear that lymphocytes are involved in both central and peripheral inflammation^26,27^. Further, platelet-lymphocyte aggregates are associated with increased thrombosis risk^28^, and venous thrombosis is a cause of sudden death in PD^29^, suggesting targeting the Pink1/Parkin pathway in the periphery has therapeutic potential.

## Results

### Pink1/Parkin deficiency modulates both glycolysis and oxidative metabolism of PBMCs.

**Figure 1:**
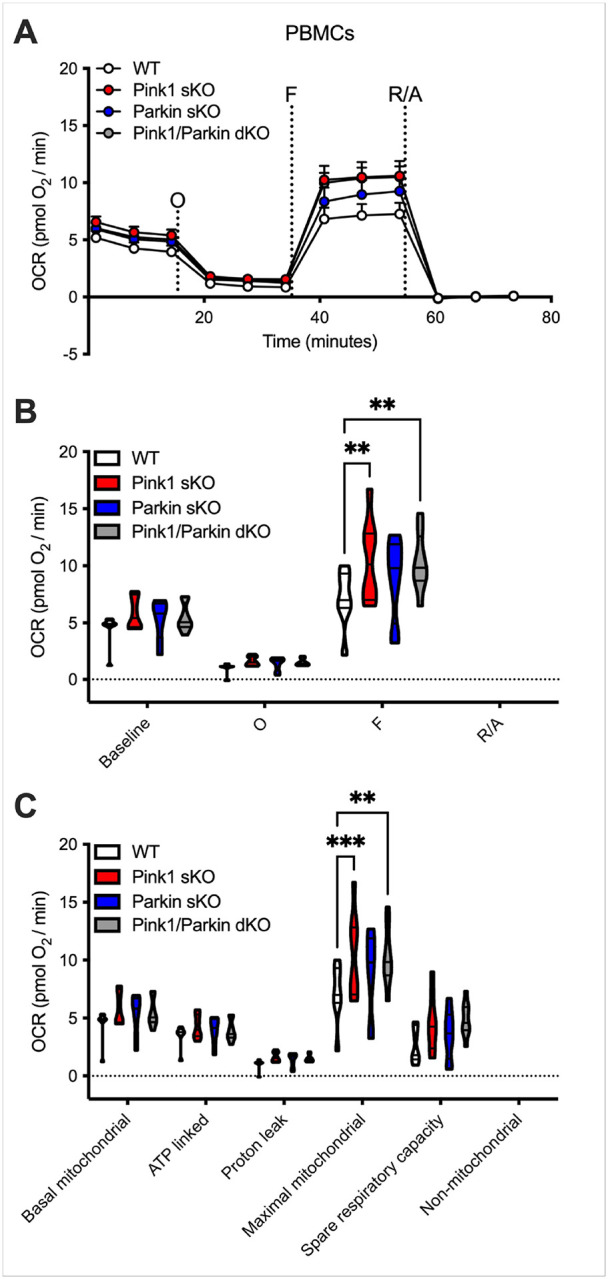
Elevated mitochondrial respiration in PBMCs isolated from Pink1 sKO and Pink1/Parkin dKO rats at 5–6 months. (A) Graphical representation of the OCR responses over time and (B) quantification of the mean OCR in A; sequential injections are indicated as O (the ATP synthase inhibitor oligomycin), F (the ATP synthesis uncoupler FCCP), R/A (a mixture of the complex I and III inhibitors rotenone and antimycin A, respectively). (C) Mitochondrial respiratory parameters calculated from the OCR shown for basal mitochondrial respiration (baseline minus R/A), ATP linked respiration (baseline minus O), proton leak (O minus R/A), maximal mitochondrial respiration (F minus R/A), and spare respiratory capacity (F minus baseline). Two-way ANOVA with Tukey’s multiple comparisons test was used to determine statistical significance (*p* < 0.01**, 0.001***; *n = 7 (WT) and 8 (Pink1 sKO, Parkin sKO, Pink1/Parkin dKO)*.

**Figure 2:**
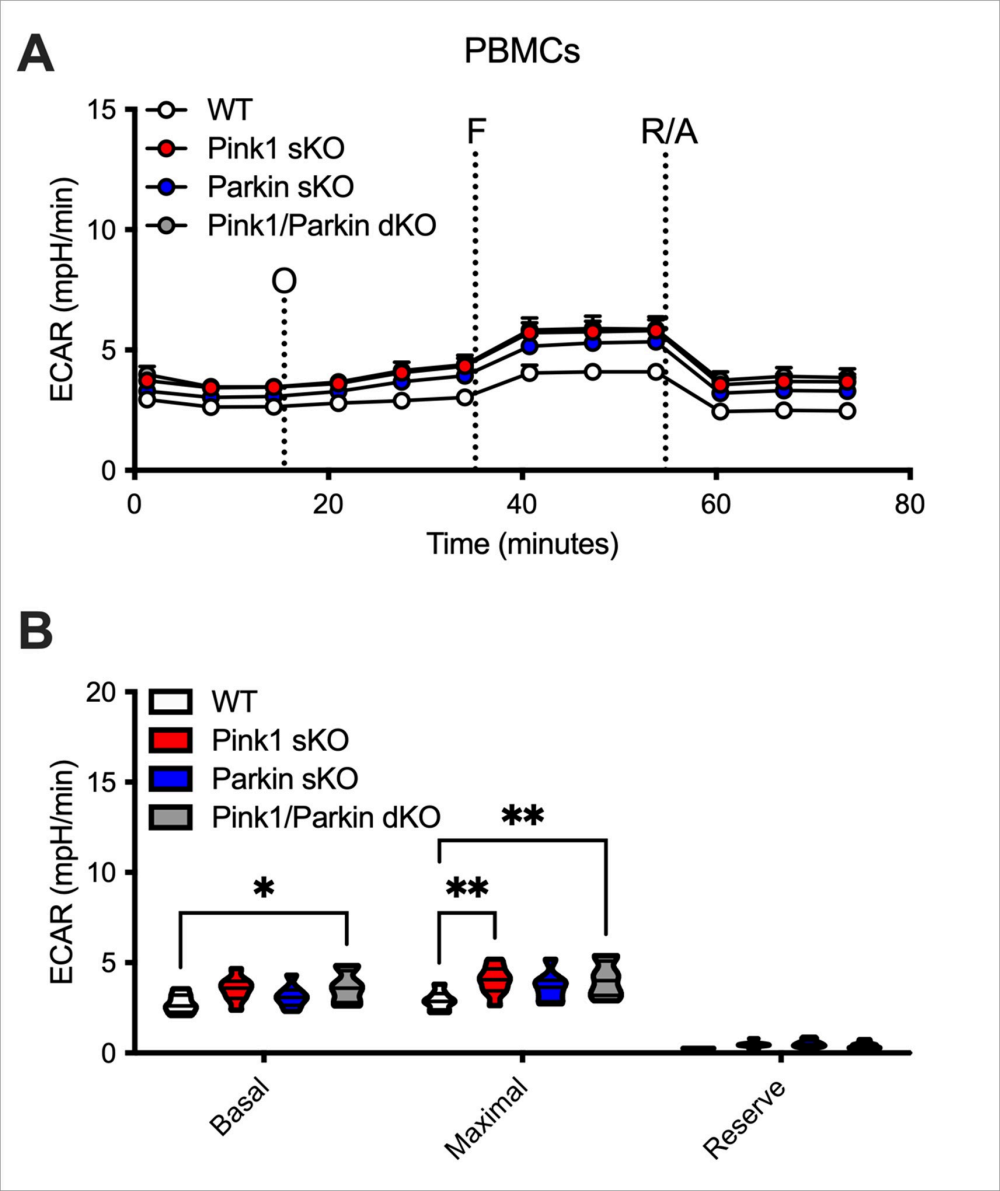
Increased glycolytic function in PBMCs isolated from Pink1 sKO and Pink1/Parkin dKO rats at 5–6 months. (A) Graphical representation of the ECAR responses over time and (B) quantification of the mean OCR in A for basal glycolysis (baseline) and maximal glycolysis (O), as well as glycolytic reserve (O minus baseline). Data after FCCP and R/A injection were not used for ECAR analysis. Two-way ANOVA with Tukey’s multiple comparisons test was used to determine statistical significance (*p* < 0.05*, 0.01**; *n* = 7 (WT) and 8 (Pink1 sKO, Parkin sKO, Pink1/Parkin dKO).

**Figure 3:**
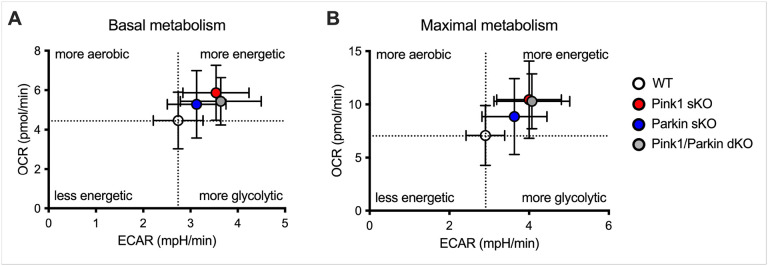
Loss of Pink1/Parkin increases the metabolic phenotype of PBMCs from rats at 5–6 months. Overall energetic phenotype of the PBMCs shown as OCR as a function of ECAR for (A) basal and (B) maximal metabolism.

While no significant differences in the number of PBMCs isolated per mL of whole blood were uncovered (**Supplementary Figure S1O**), we investigated whether Pink1 and/or Parkin deficiency could modulate the metabolic phenotype displayed by the PBMCs. Mitochondrial respiration (OCR) and glycolysis (ECAR) were measured in PBMCs from WT, Pink1 sKO, Parkin sKO, and Pink1/Parkin dKO rats using the Seahorse Analyzer. Sequential additions of an ATP synthase inhibitor (O), ATP synthesis uncoupler (F), and mixture of complex I and III inhibitors (R/A) allowed determination of basal mitochondrial respiration, ATP production-linked rate, proton leakage, maximal mitochondrial respiration, spare respiratory capacity, and non-mitochondrial respiration. No significant mitochondrial respiratory alterations were uncovered in PBMCs from Parkin sKO rats (Fig. 1). In contrast, PBMCs from Pink1 sKO and Pink1/Parkin dKO rats exhibit increased respiration following uncoupling of ATP synthesis (Fig. 1A, B) and elevated maximal mitochondrial respiration compared to those from WT rats (Fig. 1C). The glycolytic function of PBMCs from Parkin sKO rats is unaltered compared to those from WT rats (Fig. 2). While PBMCs from Pink1/Parkin dKO rats have elevated basal and maximal glycolytic function, PBMCs from Pink1 sKO rats only show increased basal glycolytic function (Fig. 2B). The cell energy phenotype of PBMCs was determined by plotting ECAR (glycolysis) as a function of OCR (mitochondrial respiration) revealing that under basal and maximal conditions PBMCs from Pink1- and/or Parkin-deficient rats are more energetic overall (more aerobic and glycolytic) as compared to those from WT rats (Fig. 3). Thus, the Pink1/Parkin pathway is important for metabolic regulation of PBMCs in rats, and this may be driven by contributions from Pink1 > Parkin. This is significant since PBMC metabolism is altered in several disease conditions, including PD^30–32^.

### Pink1/Parkin deficiency alters the profile of circulating lymphocytes in the peripheral blood.

**Figure 4:**
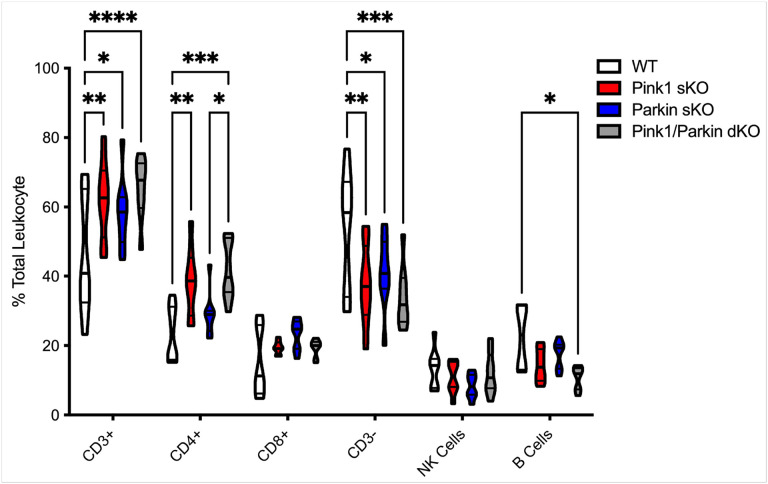
Altered levels of lymphocyte subsets circulating in Pink1-Parkin deficient rats at 5–6 months. PBMCs were isolated from WT, Pink1 sKO, Parkin sKO, and Pink1/Parkin dKO rats at 5–6 months of age and processed for flow cytometric analysis. Data shown as truncated violin plots with median and quartiles indicated by black horizontal lines. Two-way ANOVA with Tukey’s multiple comparisons test was used to determine statistical significance (*p*< 0.05*, 0.01**, 0.001***, 0.0001****; *n* = 8 per genotype).

We employed a validated flow cytometric panel^33^ to characterize the major leukocyte subsets circulating in the peripheral blood using PBMCs isolated from WT, Pink1 sKO, Parkin sKO, and Pink1/Parkin dKO rats. Using antibodies targeted against a range of surface antigens our gating strategy first removed debris and dead cells (LIVE-DEAD stain), cell clumps (FSC-H and FSC-W; exclude doublets and clustered cells), or CD45- cells (excludes erythrocytes and plasma cells). This resulted in a population of live single leukocytes which were then sequentially separated, first for T lymphocytes by CD3 which were further delineated by CD4 and CD8, and then NK cells were identi ed by CD161 and B cells by CD45R. Changes in the circulating leukocytes, specifically increased numbers of CD3 + T cells and decreased numbers of CD3- T cells were found due to loss of Pink1 and/or Parkin (Fig. 4). CD4 + T cells were found to be elevated in Pink1 sKO and Pink1/Parkin dKO rats compared to WT rats (Fig. 4). Pink1/Parkin dKO rats showed increased numbers of CD4 + T cells compared to Parkin sKO rats (Fig. 4). Reduced numbers of B cells were found in Pink1/Parkin dKO rats than in WT rats (Fig. 4). Loss of the Pink1-Parkin pathway modulates the circulating lymphocyte populations in rats, important as peripheral lymphocytes exhibit substantial quantitative and qualitative changes in PD^27^.

### Increased platelet-T cell aggregates due to Pink1/Parkin deficiency.

**Figure 5:**
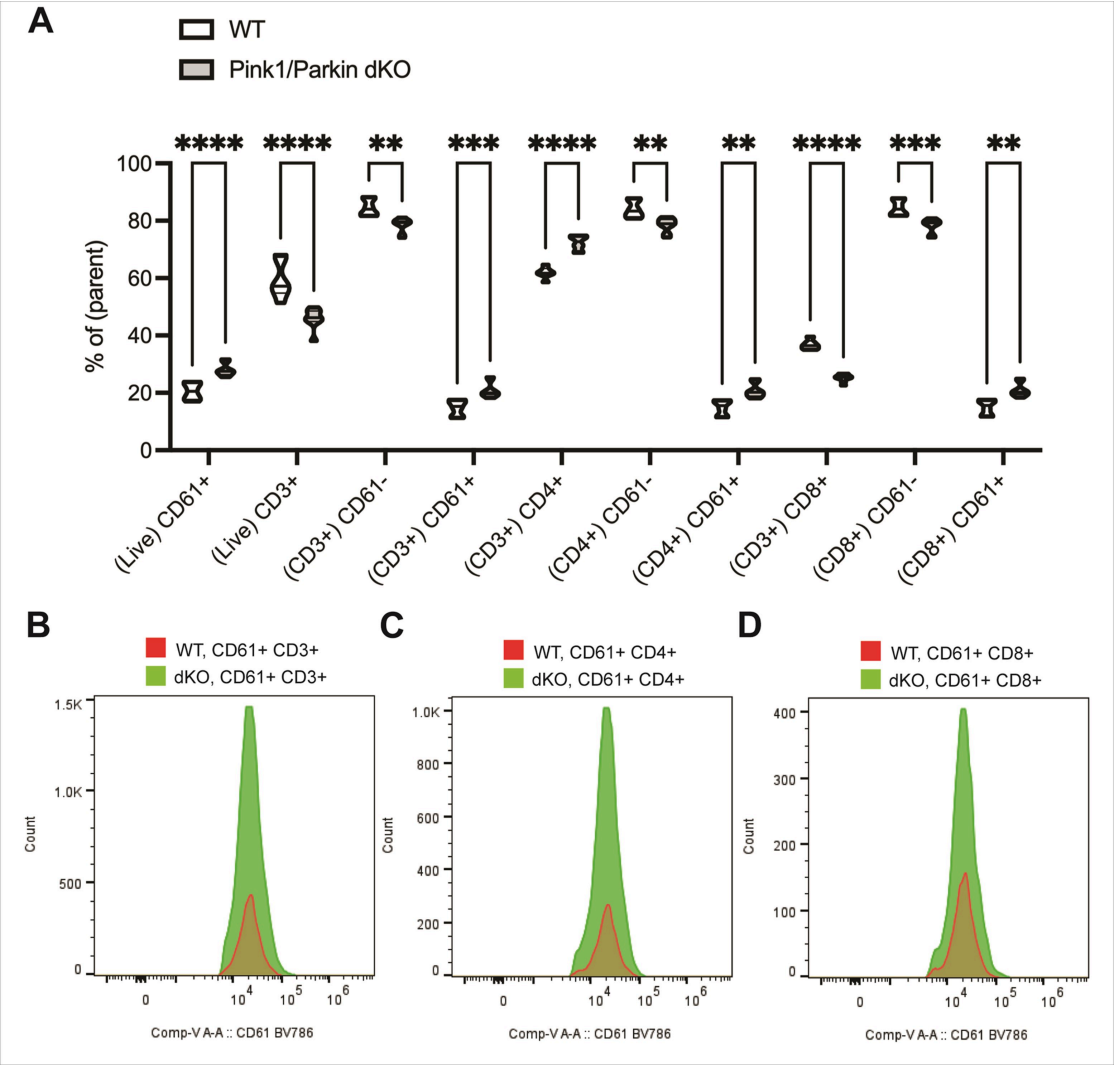
Increased platelet-T cell aggregates in Pink1/Parkin dKO rats at 5–6 months. Whole blood was isolated from WT and Pink1/Parkin dKO rats at 5–6 months of age and processed for flow cytometric analysis. (A) Data shown as truncated violin plots with median and quartiles indicated by black horizontal lines. Two-way ANOVA with Sidak’s multiple comparisons test was used to determine statistical significance (*p* < 0.001***, 0.0001****; *n* = 6 (WT) and 8 (Pink1/Parkin dKO)). Representative histograms of the counts for (B) CD61+CD3+, (C) CD61+CD4+, and (D) CD61+CD8+ populations in WT and Pink1/Parkin dKO rats.

Platelet-lymphocyte aggregates are associated with increased thrombosis risk^28^, and venous thrombosis is a cause of sudden death in PD^29^. We report increased platelet counts (number of CD61 + cells) in Pink1/Parkin dKO rats at 5–6 months (Fig. 5). Therefore, we hypothesized that platelet-T cell aggregates may be increased in these rats. Whole blood samples were gated based on surface receptor expression for each T cell subset and were further gated based on forward scatter vs side scatter to better define the population. Platelet-CD3 + T cell aggregates (CD3 + CD61 + population) were increased in Pink1/Parkin dKO rats compared to WT rats (Fig. 5). Platelet-CD4 + and platelet-CD8 + T cell aggregates (CD4 + CD61 + and CD8 + CD61 + populations, respectively) were each increased in Pink1/Parkin dKO rats compared to control WT rats (Fig. 5). Although we found increased CD3 + T cells as a percentage of total leukocytes in isolated PBMCs (Fig. 4), we found decreased CD3 + T cells as a percentage of live cells in whole blood from Pink1/Parkin dKO rats (Fig. 5). As a percentage of live CD3 + T cells in whole blood we found increased CD4 + T cells (Fig. 5), similar to our findings of increased CD4 + T cells as a percentage of total leukocytes in isolated PBMCs from Pink1/Parkin dKO rats (Fig. 4). Further, while no significant change in CD8 + T cells was uncovered as a percentage of total leukocytes in isolated PBMCs (Fig. 4), we did uncover a significant decrease in the number of CD8 + T cells as a percentage of live CD3 + T cells in whole blood from Pink1/Parkin dKO rats (Fig. 5).

## Discussion

We previously reported that maximal mitochondrial respiration is impaired in PBMCs from Pink1/Parkin dKO rats at 12 months of age^25^. Here, we compared the metabolic alterations in PBMCs derived from Pink1 sKO, Parkin sKO, and Pink1/Parkin dKO rats at 5–6 months of age to uncover the effect of age as well as role that Pink1 and Parkin play individually. We found that PBMCs from Pink1/Parkin dKO rats at 5–6 months exhibit elevated maximal mitochondrial respiration, revealing an age-dependence on mitochondrial functional alterations. Similar to Pink1/Parkin dKO rats, we found that PBMCs from Pink1 sKO rats also exhibit elevated maximal mitochondrial respiration; however, mitochondrial respiration parameters in PBMCs from Parkin sKO rats are unaltered, suggesting it is Pink1 driving this effect. PBMCs from younger (2–2.5 months old) Pink1 sKO rats were previously shown to exhibit elevated mitochondrial respiration compared to PBMCs from WT controls^34^. We also found that maximal glycolysis is increased in PBMCs from Pink1 sKO and Pink1/Parkin dKO rats, while basal glycolysis is higher only in Pink1/Parkin dKO rats. Again, PBMCs from Parkin sKO rats show glycolytic function comparable to WT. In younger (2–2.5 months old) Pink1 sKO rats, PBMCs were shown to exhibit elevated basal glycolysis compared to PBMCs from WT controls^34^. The lack of basal glycolytic changes in Pink1 sKO rats in our study could be due to age-related effects on PBMC glycolytic function or due to sex effects since we studied only males, while the previous work was from males and females compiled together. Overall, the energetic phenotype of PBMCs from the genetic PD model rats was increased (Pink1/Parkin dKO = Pink1 sKO > Parkin sKO > WT), indicating they are more aerobic and glycolytic. Bioenergetics have been studied in PBMCs from PD patients and point to increased energetics in general. In one study no differences in mitochondrial respiration were uncovered between PBMCs from control and PD patients, a marked elevation in glycolysis was found in PD patient PBMCs^30^. However, in another study while mitochondrial basal respiration was normal, maximal respiration and spare respiratory capacity were in increased in PBMCs from PD patients, and this correlated with clinical disease measures^31^. Consistent with this, another study showed elevated mitochondrial activity in PBMCs from PD patients^32^.

Our prior study using isolated PBMCs revealed increased CD4 + T cells and decreased CD8 + T cells in Pink1/Parkin dKO rats at 12 months^25^. To assess the effects of Pink1 and Parkin alone as well as age on the circulating lymphocyte populations in rats, we isolated PBMCs from Pink1 sKO, Parkin sKO, and Pink1/Parkin dKO rats at 5–6 months of age, an age when Pink1 sKO and Pink1/Parkin dKO rats exhibit motor symptoms^17,22,24^. We found that the percentage of CD3 + T cells was higher in Pink1 sKO, Parkin sKO, and Pink1/Parkin dKO rats as compared to WT controls. In particular, the levels of CD3 + CD4 + T cells were elevated in Pink1 sKO and Pink1/Parkin dKO rats compared to WT rats (also elevated in Pink1/Parkin dKO rats compared to Parkin sKO rats). T lymphocytes are key modulators of both humoral and cellular adaptive immune responses, and their role in PD is increasingly appreciated. While some report an increased percentage of CD4 + T cells in PD patients^35,36^, others have found decreased numbers of circulating CD4 + T cells^37–40^ or insignificant changes^41–43^. While no significant changes in the circulating CD8 + T cells were uncovered in the isolated rat PBMCs, when we assess whole blood from Pink1/Parkin dKO rats we found decreased numbers of CD8 + T cells compared to WT rats. The percentage of CD8 + T cells circulating in PD patients in the reported studies is quite heterogeneous. In PD patients, the levels of circulating CD8 + T cells have been found to be decreased^39,44^, increased^39,45,46^, or not changing^40,42,43^. The variations in reported lymphocyte pathology in PD patients could be associated with various factors, including age, sex, ethnicity, disease duration, and disease severity. Further, the influence of medication cannot be overlooked in PD patients. Additionally, studies of T cells in genetic PD patients and stratification of such findings by the genetic component will aid our understanding of the role of Pink1 and Parkin in human PD.

While no changes in B cell numbers were found in Pink1/Parkin dKO rats at 12 months^25^, at 6 months, we found reduced numbers of B cells in Pink1/Parkin dKO compared to WT rats. B cells were found to be reduced in transgenic α-synuclein mouse models of PD, where it was shown regulatory B cells play a protective role, potentially attenuating inflammation and dopaminergic neuron loss^47^. It is increasingly appreciated that B cells are reduced in PD^37,48–50^ and a more pro-inflammatory state in the B cell compartment has been consistently described^39,47,50,51^. B lymphocytes perform a variety of roles as part of the adaptive immune system and recent evidence suggests B cells are likely to interact with the central nervous system in complex ways via the meningeal lymphatic system^52,53^ and via egress through channels in the skull bone marrow^54,55^. These findings suggest B cells may play a role in inflammation in neurodegenerative diseases, including PD, both peripherally and centrally, warranting further study.

We previously reported a significant elevation in the number of platelets in the blood of Pink1/Parkin dKO rats at 9 and 12 months of age^25^. Here, we found that the number of CD61 + platelets are increased in Pink1/Parkin dKO rats at 6 months of age. Platelets, small non-nucleated blood cells, are gaining recognition for novel functions beyond their traditional role in hemostasis and wound closure, revealing them to be important players during immune responses and tissue remodeling. Further, platelet dysfunction is linked to several pathologies, including neurodegeneration. Platelet structural and functional alterations are evident in PD. Platelets from PD patients show reduced mitochondrial electron transport chain (ETC) complex activities^56,57^, lower vesicular monoamine transporter 2 mRNA^58^, and decreased glutamate uptake^59^. Contradictory findings have been reported regarding platelet counts with some reports for decreased number^60–62^ and others of unchanged counts^63^ in PD patients; however, increases in mean platelet volume are found^60^. Platelet aggregation induced by agonists (ADP and epinephrine) was significantly decreased in PD patients, and exogenous human α-synuclein acts as a mild platelet antiaggregant *in vitro*^64^. The highest concentration of α-synuclein per mg of cellular protein in the blood is found in platelets^65,66^. In addition to α-synuclein, platelets express various other PD-relevant proteins, including tyrosine hydroxylase^67^, dopamine transporter^68^, Pink1^69^, and Parkin^70^. Of note, we previously reported elevated levels of plasma α-synuclein in the Pink1/Parkin dKO rats^25^.

The role of platelets in the regulation of immune cells such as T cells is becoming increasingly appreciated. Platelets participate in inflammation by producing pro-inflammatory mediators, which are stored in their vesicles (granules) prior to release^71,72^. Platelets communicate with immune cells, including T cells, via release of such mediator as well as through release of neurotransmitters (e.g., serotonin, epinephrine, dopamine, and histamine)^73–75^. Additionally, platelets and T cells can form direct contacts with each other. In fact, platelet-T cell aggregates correlate with markers of platelet aggregation, immune activation, and disease progression^28,76–79^. The role of platelet-T cell aggregates during PD and in animal models of PD remain underexplored. Thus, we assessed platelet-T cell aggregation in the Pink1/Parkin dKO rat model. We found that Pink1/Parkin dKO rats have a higher percentage of platelet-CD3 + T cell aggregates, including both increased platelet-CD4 + T cell and platelet-CD8 + T cell aggregation. Platelet-lymphocyte aggregates are associated with increased thrombosis risk, and venous thrombosis is a cause of sudden death in PD^28,29^. Thus, additional studies will inform on the contribution of platelet-lymphocyte aggregation to disease progression in the Pink1/Parkin dKO rat model and we believe studies to assess platelet-lymphocyte aggregation in PD patients are warranted.

In summary, we describe changes in the circulating leukocytes, specifically increased numbers of CD4 + T cells and decreased numbers of CD8 + T cells and B cells in Pink1/Parkin dKO rats. Increased CD4 + T cells were found in Pink1 sKO rats, suggesting Pink1 might drive this effect. In contrast to our findings of reduced PBMC mitochondrial respiration at 12 months^25^, we found elevated mitochondrial respiration at 6 months in dKO rats, which appears to be largely driven by loss of Pink1. Further, we uncovered elevated PBMC glycolytic function, which taken together reveals an immunometabolic shift towards a more energetic phenotype is induced by loss of Pink/Parkin. Platelet numbers were elevated, and platelet-T cell aggregation was increased in Pink1/Parkin dKO rats, involving both CD4 + and CD8 + T cells. These findings further support a role for the peripheral immune system and highlight a previously unappreciated role for platelet-T cell aggregates in Pink1/Parkin-linked PD pathogenesis.

## Methods

### Animal subjects

Rats with targeted disruption of the *Park6* gene (Pink1 single knockout, sKO), the *Park2* gene (Parkin sKO), and the background strain (Long Evans Hooded) were obtained from SAGE Labs (now Inotiv)^17^. Pink1/Parkin double KO (dKO) rats were generated in our lab from the sKO rats as described previously^24,25^. The rats were maintained on a 12-hour light/dark cycle in a temperature-controlled environment with free access to standard rat chow and water. Experimental rats were bred in-house in accordance with institutionally approved breeding protocols. All breeding and experimental procedures described herein were approved by the University of Nebraska Medical Center Institutional Animal Care and Use Committee and carried out in accordance with approved protocols and regulations. Study methods within were conducted and are reported in accordance with the ARRIVE guidelines. Rats (male, 5–6 months of age) were rendered unconscious by 5% iso urane inhalation using an anesthetic chamber, body weight was recorded, and the unconscious rats were euthanized by decapitation using a guillotine, following decapitation trunk blood was collected using vacutainer tubes containing 7.2 mg K_2_EDTA and the tissues were quickly removed and weighed. The characteristics of Pink1/Parkin deficient and control rats are presented in **Supplementary Data S1** and **S2**. The body weights of Pink1/Parkin dKO rats were significantly more than Pink1 sKO and WT rats, but not than Parkin sKO rats (**Figure S1A**). The body weights of Pink1 sKO and Parkin sKO rats were not significantly different from each other or from wild-type (WT) rats (**Figure S1A**). Because of the significant difference in body weights for Pink1/Parkin dKO rats compared to Pink1 sKO and WT rats, in addition to absolute weight of tissues (**Figure S2**), it is important to consider tissue weights normalized to body weight (**Figure S1B-N**). The brain weight was significantly lower in Parkin sKO rats than Pink1 sKO rats (**Figure S1B**). While Pink1 sKO and Pink1/Parkin dKO rats were comparable, their heart weights were significantly higher than Parkin sKO and WT rats (**Figure S1C**). Pink1 sKO rats had significantly heavier liver (**Figure S1D**), spleen (**Figure S1E**), and testis (**Figure S1F**) than Parkin sKO, Pink1/Parkin dKO, and WT rats, which were all similar to each other. Parkin sKO rats had significantly lighter kidneys than Pink1 sKO, Pink1/Parkin dKO, and WT rats, which were comparable to each other (**Figure S1G**). No significant differences were found for lung (**Figure S1H**), quadricep (**Figure S1I**), and hamstring (**Figure S1J**). Pink1 sKO and Parkin sKO rats had significantly increased anterior tibialis mass than WT rats (**Figure S1K**). The extensor digitorum longus was heavier in Parkin sKO rats than in Pink1 sKO, Pink1/Parkin dKO, and WT rats (**Figure S1L**). The gastrocnemius weight in Pink1 sKO and Pink1/Parkin dKO rats was significantly higher than in Parkin sKO and WT rats (**Figure S1M**). WT rats have significantly lighter soleus than Pink1 sKO, Parkin sKO, and Pink1/Parkin dKO rats (**Figure S1N**). The differences that we uncovered between the genotypes reveals tissue-specific effects of loss of Pink1 and/or Parkin.

### Isolation of PBMCs

Whole blood was centrifuged at 900 × g for 20 min at RT to separate erythrocytes, plasma, and the buffy coat. The buffy coat was removed, diluted with 1x PBS, and overlayed on Ficoll-Hypaqe prior to centrifugation at 400 × g for 30 min at RT. The PBMC layer was collected, diluted with 1x PBS, and centrifuged at 500 × g for 7 min at RT. The resulting pellet was lysed with 1x RBC lysis buffer, diluted with 1x PBS, and centrifuged at 400 × g for 7 min at RT. The PBMC pellet was resuspended in 1x PBS and counted using a Beckman Coulter z1 particle counter. PBMCs were cryopreserved in Fetal Bovine Serum with 10% DMSO and stored in liquid nitrogen. Cells were thawed within 6 weeks of isolation and underwent bioenergetic and flow cytometric assessments.

### Bioenergetic assays

Oxygen consumption rates (OCR) and extracellular acidi cation rates (ECAR) were measured at 37°C on the Seahorse XFe96 Extracellular Flux Analyzer using 6 technical replicate wells for each independent biological replicate. PBMCs were plated at 750,000 cells/well in XF assay medium containing 4 mM L-glutamine and 25 mM glucose. Three baseline measurements of OCR and ECAR were recorded prior to sequential injection of oligomycin (O, 3.5 μM, ATP synthase inhibitor), carbonyl cyanide 4-(tri uoromethoxy)phenylhydrazone (FCCP, F, 1 μM, mitochondrial oxidative phosphorylation uncoupler), rotenone (R, 14 μM, complex I inhibitor) and antimycin A (A, 14 μM, complex III inhibitor). The Seahorse Wave version 2.6.1 software package was used for data calculation as described previously^25^.

### Flow cytometry (PBMCs)

PBMCs were washed and stained with Live/Dead Fixable Blue Dead Cell Stain (Invitrogen) in PBS. Cells were then washed and blocked with anti-CD32 (1:50, 550270, BD Biosciences) to prevent Fc-mediated non-specific binding. After blocking, cells were stained with the following antibodies: BV605-conjugated anti-CD45 (1:100, 740371, BD Biosciences), APC-conjugated anti-CD3 (1:100, 130.102–679, Miltenyi Biotec), V450-conjugated anti-CD4 (1:100, 561579, BD Biosciences), BUV805-conjugated anti-CD8a (1:100, 741971, BD Biosciences), FITC-conjugated anti-CD161 (1:100, 205608, BioLegend), and BB700-conjugated anti-B220 CD45R (1:100, 745880, BD Biosciences) in staining buffer containing 1x PBS, 1% Bovine Serum Albumin (BSA), and 0.1% Sodium Azide at 4°C for 30 minutes in the dark. Cells were then washed with staining buffer and fixed in a buffer containing 1x PBS and 4% paraformaldehyde. Flow cytometric compensation was performed using OneComp eBeads and analyzed using a BD LSRFortessa X50 (BD Biosciences). Gating strategy, controls, and data for each rat provided as **Supplementary Data S3**.

### Flow cytometry (whole blood)

Whole blood was stained with Live/Dead Fixable Blue Dead Cell Stain (Invitrogen) in 1x PBS with 1% BSA. All centrifugation steps were carried out at 1,200 × g for 5 minutes to ensure platelet collection. The cells and platelets were then washed and blocked with anti-CD32 to prevent Fc-mediated non-specific binding. After blocking, cells were re-suspended in 100uL 1x PBS with 1% BSA and stained with the following antibodies: BV786-conjugated anti-CD61 (1:30, 744460, BD Biosciences), APC-conjugated anti-CD3 (1:30, 130–102-679, Miltenyi Biotec), V450-conjugated anti-CD4 (1:30, 561579, BD Biosciences), BUV805-conjugated anti-CD8a (1:30, 741971, BD Biosciences), and 50 μL Brilliant Stain Buffer (50:150, 563794, BD Horizon) at 4°C for 45 minutes in the dark. Cells were then washed in 1x PBS with 1% BSA and fixed in a buffer containing 1x PBS and 4% paraformaldehyde. Flow cytometric compensation was performed using OneComp eBeads and analyzed using a BD LSRFortessa X50 (BD Biosciences). Gating strategy, controls, and data for each rat provided as **Supplementary Data S4**.

### Statistical analysis

Descriptive data are presented as violin plots showing the median and quartiles. Group comparisons were conducted using one-way analysis of variance (ANOVA) or two-way ANOVA with Tukey’s multiple comparisons test or Sidak’s multiple comparisons test, as appropriate for variance of data. Statistical analysis was performed using GraphPad Prism version 10.1.1 (GraphPad Software, San Diego, CA).

## Data Availability

The data supporting the findings in this study are available within the article and/or its supplementary material.

## References

[1] Kalia L. V. & Lang A. E. Parkinson’s disease. Lancet 386, 896–912, doi:10.1016/S0140-6736(14)61393-3 (2015). 25904081

[2] Behari M. Parkinson’s disease. Ann Indian Acad Neurol 14, S2–6, doi:10.4103/0972-2327.83083 (2011). 21847323 PMC3152168

[3] Bove J. & Perier C. Neurotoxin-based models of Parkinson’s disease. Neuroscience 211, 51–76, doi:10.1016/j.neuroscience.2011.10.057 (2012). 22108613

[4] Tanner C. M. Rotenone, paraquat, and Parkinson’s disease. Environ Health Perspect 119, 866–872, doi:10.1289/ehp.1002839 (2011). 21269927 PMC3114824

[5] Faull R. L. & Laverty R. Changes in dopamine levels in the corpus striatum following lesions in the substantia nigra. Exp Neurol 23, 332–340, doi:10.1016/0014-4886(69)90081-8 (1969). 5767257

[6] Hsieh T. H., Chen J. J., Chen L. H., Chiang P. T. & Lee H. Y. Time-course gait analysis of hemiparkinsonian rats following 6-hydroxydopamine lesion. Behav Brain Res 222, 1–9, doi:10.1016/j.bbr.2011.03.031 (2011). 21435355

[7] Pickrell A. M. & Youle R. J. The roles of PINK1, parkin, and mitochondrial fidelity in Parkinson’s disease. Neuron 85, 257–273, doi:10.1016/j.neuron.2014.12.007 (2015). 25611507 PMC4764997

[8] Dawson T. M., Ko H. S. & Dawson V. L. Genetic animal models of Parkinson’s disease. Neuron 66, 646–661, doi:10.1016/j.neuron.2010.04.034 (2010). 20547124 PMC2917798

[9] McLelland G. L., Soubannier V., Chen C. X., McBride H. M. & Fon E. A. Parkin and PINK1 function in a vesicular trafficking pathway regulating mitochondrial quality control. EMBO J 33, 282–295, doi:10.1002/embj.201385902 (2014). 24446486 PMC3989637

[10] Tanaka A. Proteasome and p97 mediate mitophagy and degradation of mitofusins induced by Parkin. J Cell Biol 191, 1367–1380, doi:10.1083/jcb.201007013 (2010). 21173115 PMC3010068

[11] Xiao B. Reactive oxygen species trigger Parkin/PINK1 pathway-dependent mitophagy by inducing mitochondrial recruitment of Parkin. J Biol Chem 292, 16697–16708, doi:10.1074/jbc.M117.787739 (2017). 28848050 PMC5633131

[12] Narendra D., Tanaka A., Suen D. F. & Youle R. J. Parkin is recruited selectively to impaired mitochondria and promotes their autophagy. J Cell Biol 183, 795–803, doi:10.1083/jcb.200809125 (2008). 19029340 PMC2592826

[13] Narendra D. P. PINK1 is selectively stabilized on impaired mitochondria to activate Parkin. PLoS Biol 8, e1000298, doi:10.1371/journal.pbio.1000298 (2010). 20126261 PMC2811155

[14] Tansey M. G. & Romero-Ramos M. Immune system responses in Parkinson’s disease: Early and dynamic. Eur J Neurosci 49, 364–383, doi:10.1111/ejn.14290 (2019). 30474172 PMC6391192

[15] Kannarkat G. T., Boss J. M. & Tansey M. G. The role of innate and adaptive immunity in Parkinson’s disease. J Parkinsons Dis 3, 493–514, doi:10.3233/JPD-130250 (2013). 24275605 PMC4102262

[16] Kustrimovic N., Marino F. & Cosentino M. Peripheral Immunity, Immunoaging and Neuroinflammation in Parkinson’s Disease. Curr Med Chem 26, 3719–3753, doi:10.2174/0929867325666181009161048 (2019). 30306855

[17] Dave K. D. Phenotypic characterization of recessive gene knockout rat models of Parkinson’s disease. Neurobiol Dis 70, 190–203, doi:10.1016/j.nbd.2014.06.009 (2014). 24969022

[18] de Haas R. To be or not to be pink(1): contradictory findings in an animal model for Parkinson’s disease. Brain Commun 1, fcz016, doi:10.1093/braincomms/fcz016 (2019). 31667474 PMC6798789

[19] Grant L. M. Evidence for early and progressive ultrasonic vocalization and oromotor deficits in a PINK1 gene knockout rat model of Parkinson’s disease. J Neurosci Res 93, 1713–1727, doi:10.1002/jnr.23625 (2015). 26234713 PMC4575652

[20] Creed R. B. Basal and Evoked Neurotransmitter Levels in Parkin, DJ-1, PINK1 and LRRK2 Knockout Rat Striatum. Neuroscience 409, 169–179, doi:10.1016/j.neuroscience.2019.04.033 (2019). 31029729 PMC6559826

[21] Villeneuve L. M., Purnell P. R., Boska M. D. & Fox H. S. Early Expression of Parkinson’s Disease-Related Mitochondrial Abnormalities in PINK1 Knockout Rats. Mol Neurobiol 53, 171–186, doi:10.1007/s12035-014-8927-y (2016). 25421206 PMC4442772

[22] DeAngelo V. M., Hilliard J. D. & McConnell G. C. Dopaminergic but not cholinergic neurodegeneration is correlated with gait disturbances in PINK1 knockout rats. Behav Brain Res 417, 113575, doi:10.1016/j.bbr.2021.113575 (2022). 34534596

[23] Kelm-Nelson C. A. Pink1(−/−) rats are a useful tool to study early Parkinson disease. Brain Commun 3, fcab077, doi:10.1093/braincomms/fcab077 (2021). 33928251 PMC8066864

[24] Stauch K. L. Applying the RatWalker System for Gait Analysis in a Genetic Rat Model of Parkinson’s Disease. J Vis Exp, doi:10.3791/62002 (2021). PMC11635613 33522500

[25] Lamberty B. G. Parkinson’s disease relevant pathological features are manifested in male Pink1/Parkin deficient rats. Brain Behav Immun Health 31, 100656, doi:10.1016/j.bbih.2023.100656 (2023). 37484197 PMC10362548

[26] Zhang Z. Abnormal immune function of B lymphocyte in peripheral blood of Parkinson’s disease. Parkinsonism Relat Disord 116, 105890, doi:10.1016/j.parkreldis.2023.105890 (2023). 37839276

[27] Contaldi E., Magistrelli L. & Comi C. T Lymphocytes in Parkinson’s Disease. J Parkinsons Dis 12, S65–S74, doi:10.3233/JPD-223152 (2022). 35253782 PMC9535550

[28] Meikle C. K. Platelet-T cell aggregates in lung cancer patients: Implications for thrombosis. PLoS One 15, e0236966, doi:10.1371/journal.pone.0236966 (2020). 32776968 PMC7416940

[29] Afsin E., Cosgun Z., Kurul R. & Turkoglu S. A. The incidence of deep venous thrombosis in Parkinson’s disease. Neurol Res 45, 1050–1054, doi:10.1080/01616412.2023.2257441 (2023). 37699515

[30] Smith A. M. Mitochondrial dysfunction and increased glycolysis in prodromal and early Parkinson’s blood cells. Mov Disord 33, 1580–1590, doi:10.1002/mds.104 (2018). 30294923 PMC6221131

[31] Schirinzi T. Pattern of Mitochondrial Respiration in Peripheral Blood Cells of Patients with Parkinson’s Disease. Int J Mol Sci 23, doi:10.3390/ijms231810863 (2022). PMC9506016 36142777

[32] Gevezova M., Kazakova M., Trenova A. & Sarafian V. YKL-40 and the Cellular Metabolic Profile in Parkinson’s Disease. Int J Mol Sci 24, doi:10.3390/ijms242216297 (2023). PMC10671493 38003487

[33] Barnett-Vanes A., Sharrock A., Birrell M. A. & Rankin S. A Single 9-Colour Flow Cytometric Method to Characterise Major Leukocyte Populations in the Rat: Validation in a Model of LPS-Induced Pulmonary Inflammation. PLoS One 11, e0142520, doi:10.1371/journal.pone.0142520 (2016). 26764486 PMC4713146

[34] Grigoruta M., Dagda R. K., Diaz-Sanchez A. G. & Martinez-Martinez A. Psychological distress and lack of PINK1 promote bioenergetics alterations in peripheral blood mononuclear cells. Sci Rep 10, 9820, doi:10.1038/s41598-020-66745-9 (2020). 32555260 PMC7300038

[35] Bas J. Lymphocyte populations in Parkinson’s disease and in rat models of parkinsonism. J Neuroimmunol 113, 146–152, doi:10.1016/s0165-5728(00)00422-7 (2001). 11137586

[36] Chen X. Evidence for Peripheral Immune Activation in Parkinson’s Disease. Front Aging Neurosci 13, 617370, doi:10.3389/fnagi.2021.617370 (2021). 33994989 PMC8119625

[37] Stevens C. H. Reduced T helper and B lymphocytes in Parkinson’s disease. J Neuroimmunol 252, 95–99, doi:10.1016/j.jneuroim.2012.07.015 (2012). 22910543

[38] Niwa F., Kuriyama N., Nakagawa M. & Imanishi J. Effects of peripheral lymphocyte subpopulations and the clinical correlation with Parkinson’s disease. Geriatr Gerontol Int 12, 102–107, doi:10.1111/j.1447-0594.2011.00740.x (2012). 21929737

[39] Yan Z. Dysregulation of the Adaptive Immune System in Patients With Early-Stage Parkinson Disease. Neurol Neuroimmunol Neuroinflamm 8, doi:10.1212/NXI.0000000000001036 (2021). PMC8299515 34301818

[40] Sun C. Abnormal subpopulations of peripheral blood lymphocytes are involved in Parkinson’s disease. Ann Transl Med 7, 637, doi:10.21037/atm.2019.10.105 (2019). 31930038 PMC6944630

[41] Rocha N. P. Reduced Activated T Lymphocytes (CD4+CD25+) and Plasma Levels of Cytokines in Parkinson’s Disease. Mol Neurobiol 55, 1488–1497, doi:10.1007/s12035-017-0404-y (2018). 28176275

[42] Schroder J. B. Immune Cell Activation in the Cerebrospinal Fluid of Patients With Parkinson’s Disease. Front Neurol 9, 1081, doi:10.3389/fneur.2018.01081 (2018). 30619041 PMC6305582

[43] Cen L. Peripheral Lymphocyte Subsets as a Marker of Parkinson’s Disease in a Chinese Population. Neurosci Bull 33, 493–500, doi:10.1007/s12264-017-0163-9 (2017). 28791571 PMC5636734

[44] Kouli A. T lymphocyte senescence is attenuated in Parkinson’s disease. J Neuroinflammation 18, 228, doi:10.1186/s12974-021-02287-9 (2021). 34645462 PMC8513368

[45] Baba Y., Kuroiwa A., Uitti R. J., Wszolek Z. K. & Yamada T. Alterations of T-lymphocyte populations in Parkinson disease. Parkinsonism Relat Disord 11, 493–498, doi:10.1016/j.parkreldis.2005.07.005 (2005). 16154792

[46] Wang P. Single-cell transcriptome and TCR profiling reveal activated and expanded T cell populations in Parkinson’s disease. Cell Discov 7, 52, doi:10.1038/s41421-021-00280-3 (2021). 34282123 PMC8289849

[47] Scott K. M. B lymphocyte responses in Parkinson’s disease and their possible significance in disease progression. Brain Commun 5, fcad060, doi:10.1093/braincomms/fcad060 (2023). 36993946 PMC10042276

[48] Gruden M. A. Immunoprotection against toxic biomarkers is retained during Parkinson’s disease progression. J Neuroimmunol 233, 221–227, doi:10.1016/j.jneuroim.2010.12.001 (2011). 21239064

[49] Kedmi M., Bar-Shira A., Gurevich T., Giladi N. & Orr-Urtreger A. Decreased expression of B cell related genes in leukocytes of women with Parkinson’s disease. Mol Neurodegener 6, 66, doi:10.1186/1750-1326-6-66 (2011). 21943286 PMC3189133

[50] Li R. Abnormal B-Cell and Tfh-Cell Profiles in Patients With Parkinson Disease: A Cross-sectional Study. Neurol Neuroimmunol Neuroinflamm 9, doi:10.1212/NXI.0000000000001125 (2022). PMC8711073 34955458

[51] Wang P. Global Characterization of Peripheral B Cells in Parkinson’s Disease by Single-Cell RNA and BCR Sequencing. Front Immunol 13, 814239, doi:10.3389/fimmu.2022.814239 (2022). 35250991 PMC8888848

[52] Louveau A. CNS lymphatic drainage and neuroinflammation are regulated by meningeal lymphatic vasculature. Nat Neurosci 21, 1380–1391, doi:10.1038/s41593-018-0227-9 (2018). 30224810 PMC6214619

[53] Zou W. Blocking meningeal lymphatic drainage aggravates Parkinson’s disease-like pathology in mice overexpressing mutated alpha-synuclein. Transl Neurodegener 8, 7, doi:10.1186/s40035-019-0147-y (2019). 30867902 PMC6396507

[54] Herisson F. Direct vascular channels connect skull bone marrow and the brain surface enabling myeloid cell migration. Nat Neurosci 21, 1209–1217, doi:10.1038/s41593-018-0213-2 (2018). 30150661 PMC6148759

[55] Kolabas Z. I. Distinct molecular profiles of skull bone marrow in health and neurological disorders. Cell 186, 3706–3725 e3729, doi:10.1016/j.cell.2023.07.009 (2023). 37562402 PMC10443631

[56] Benecke R., Strumper P. & Weiss H. Electron transfer complexes I and IV of platelets are abnormal in Parkinson’s disease but normal in Parkinson-plus syndromes. Brain 116 (Pt 6), 1451–1463, doi:10.1093/brain/116.6.1451 (1993). 8293280

[57] Haas R. H. Low platelet mitochondrial complex I and complex II/III activity in early untreated Parkinson’s disease. Ann Neurol 37, 714–722, doi:10.1002/ana.410370604 (1995). 7778844

[58] Sala G. Vesicular monoamine transporter 2 mRNA levels are reduced in platelets from patients with Parkinson’s disease. J Neural Transm (Vienna) 117, 1093–1098, doi:10.1007/s00702-010-0446-z (2010). 20665056

[59] Ferrarese C. Reduced platelet glutamate uptake in Parkinson’s disease. J Neural Transm (Vienna) 106, 685–692, doi:10.1007/s007020050189 (1999). 10907727

[60] Kocer A. Assessment of platelet indices in patients with neurodegenerative diseases: mean platelet volume was increased in patients with Parkinson’s disease. Curr Gerontol Geriatr Res 2013, 986254, doi:10.1155/2013/986254 (2013). 24382959 PMC3870626

[61] Machaczka M., Rucinska M., Skotnicki A. B. & Jurczak W. Parkinson’s syndrome preceding clinical manifestation of Gaucher’s disease. Am J Hematol 61, 216–217, doi:10.1002/(sici)1096-8652(199907)61:3<216::aid-ajh12>3.0.co;2-b (1999). 10398575

[62] Behari M. & Shrivastava M. Role of platelets in neurodegenerative diseases: a universal pathophysiology. Int J Neurosci 123, 287–299, doi:10.3109/00207454.2012.751534 (2013). 23301959

[63] Sharma P., Nag D., Atam V., Seth P. K. & Khanna V. K. Platelet aggregation in patients with Parkinson’s disease. Stroke 22, 1607–1608, doi:10.1161/01.str.22.12.1607 (1991). 1962340

[64] Acquasaliente L. Exogenous human alpha-Synuclein acts in vitro as a mild platelet antiaggregant inhibiting alpha-thrombin-induced platelet activation. Sci Rep 12, 9880, doi:10.1038/s41598-022-12886-y (2022). 35701444 PMC9198058

[65] Tashkandi H., Shameli A., Harding C. V. & Maitta R. W. Ultrastructural changes in peripheral blood leukocytes in alpha-synuclein knockout mice. Blood Cells Mol Dis 73, 33–37, doi:10.1016/j.bcmd.2018.09.001 (2018). 30195626 PMC6163077

[66] Stefaniuk C. M., Schlegelmilch J., Meyerson H. J., Harding C. V. & Maitta R. W. Initial assessment of alpha-synuclein structure in platelets. J Thromb Thrombolysis 53, 950–953, doi:10.1007/s11239-021-02607-z (2022). 34797472 PMC9117560

[67] Drozdov A. Z. & Anokhina I. P. [Activity of tyrosine hydroxylase and monoamine oxidase in human platelets during alcoholism]. Vopr Med Khim 36, 54–57 (1990). 1971467

[68] Frankhauser P. Characterization of the neuronal dopamine transporter DAT in human blood platelets. Neurosci Lett 399, 197–201, doi:10.1016/j.neulet.2006.01.062 (2006). 16490314

[69] Walsh T. G., van den Bosch M. T. J., Lewis K. E., Williams C. M. & Poole A. W. Loss of the mitochondrial kinase PINK1 does not alter platelet function. Sci Rep 8, 14377, doi:10.1038/s41598-018-32716-4 (2018). 30258205 PMC6158262

[70] Lee S. H., Du J., Hwa J. & Kim W. H. Parkin Coordinates Platelet Stress Response in Diabetes Mellitus: A Big Role in a Small Cell. Int J Mol Sci 21, doi:10.3390/ijms21165869 (2020). PMC7461561 32824240

[71] Thomas M. R. & Storey R. F. The role of platelets in inflammation. Thromb Haemost 114, 449–458, doi:10.1160/TH14-12-1067 (2015). 26293514

[72] Semple J. W., Italiano J. E. Jr. & Freedman J. Platelets and the immune continuum. Nat Rev Immunol 11, 264–274, doi:10.1038/nri2956 (2011). 21436837

[73] Blair P. & Flaumenhaft R. Platelet alpha-granules: basic biology and clinical correlates. Blood Rev 23, 177–189, doi:10.1016/j.blre.2009.04.001 (2009). 19450911 PMC2720568

[74] Goubau C., Buyse G. M., Di Michele M., Van Geet C. & Freson K. Regulated granule trafficking in platelets and neurons: a common molecular machinery. Eur J Paediatr Neurol 17, 117–125, doi:10.1016/j.ejpn.2012.08.005 (2013). 22951324

[75] Pavlovic V., Ciric M., Jovanovic V. & Stojanovic P. Platelet Rich Plasma: a short overview of certain bioactive components. Open Med (Wars) 11, 242–247, doi:10.1515/med-2016-0048 (2016). 28352802 PMC5329835

[76] Hottz E. D. Platelet activation and platelet-monocyte aggregate formation trigger tissue factor expression in patients with severe COVID-19. Blood 136, 1330–1341, doi:10.1182/blood.2020007252 (2020). 32678428 PMC7483437

[77] Sanz-Martinez M. T. High Levels of Platelet-Lymphocyte Complexes in Patients with Psoriasis Are Associated with a Better Response to Anti-TNF-alpha Therapy. J Invest Dermatol 140, 1176–1183, doi:10.1016/j.jid.2019.08.457 (2020). 31778714

[78] Nkambule B. B., Davison G. & Ipp H. Platelet leukocyte aggregates and markers of platelet aggregation, immune activation and disease progression in HIV infected treatment naive asymptomatic individuals. J Thromb Thrombolysis 40, 458–467, doi:10.1007/s11239-015-1212-8 (2015). 25899563

[79] Liang H. Higher levels of circulating monocyte-platelet aggregates are correlated with viremia and increased sCD163 levels in HIV-1 infection. Cell Mol Immunol 12, 435–443, doi:10.1038/cmi.2014.66 (2015). 25109683 PMC4496539

